# An evaluation of the species and subspecies of the genus *Salmonella* with whole genome sequence data: Proposal of type strains and epithets for novel *S. enterica* subspecies VII, VIII, IX, X and XI

**DOI:** 10.1016/j.ygeno.2021.07.003

**Published:** 2021-09

**Authors:** Madison E. Pearce, Gemma C. Langridge, A.C. Lauer, Kathie Grant, Martin C.J. Maiden, Marie A. Chattaway

**Affiliations:** a Department of Zoology, University of Oxford, Peter Medawar Building for Pathogen Research, South Parks Road, Oxford OX1 3SY, United Kingdom; b National Institute for Health Research, Health Protection Research Unit, Gastrointestinal Infections, University of Oxford, United Kingdom; c Quadram Institute Bioscience, Norwich Research Park, Norwich NR4 7UQ, United Kingdom; d Centers for Disease Control and Prevention, Enteric Diseases Laboratory Branch, 1600 Clifton RD NE, Atlanta, GA 30329, USA; e Public Health England, Gastrointestinal Bacteria Reference Unit, 61 Colindale Avenue, London NW9 5EQ, United Kingdom

**Keywords:** Salmonella, Genomics, Londinensis, Brasiliensis, Hibernicus, Essexiensis, Reptilium

## Abstract

Species and subspecies within the *Salmonella* genus have been defined for public health purposes by biochemical properties; however, reference laboratories have increasingly adopted sequence-based, and especially whole genome sequence (WGS), methods for surveillance and routine identification. This leads to potential disparities in subspecies definitions, routine typing, and the ability to detect novel subspecies. A large-scale analysis of WGS data from the routine sequencing of clinical isolates was employed to define and characterise *Salmonella* subspecies population structure, demonstrating that the *Salmonella* species and subspecies were genetically distinct, including those previously identified through phylogenetic approaches, namely: *S. enterica* subspecies *londinensis* (VII), subspecies *brasiliensis* (VIII), subspecies *hibernicus* (IX) and subspecies *essexiensis* (X). The analysis also identified an additional novel subspecies, *reptilium* (XI). Further, these analyses indicated that *S. enterica* subspecies *arizonae* (IIIa) isolates were divergent from the other *S. enterica* subspecies, which clustered together and, on the basis of ANI analysis, subspecies IIIa was sufficiently distinct to be classified as a separate species, *S. arizonae*. Multiple phylogenetic and statistical approaches generated congruent results, suggesting that the proposed species and subspecies structure was sufficiently biologically robust for routine application. Biochemical analyses demonstrated that not all subspecies were distinguishable by these means and that biochemical approaches did not capture the genomic diversity of the genus. We recommend the adoption of standardised genomic definitions of species and subspecies and a genome sequence-based approach to routine typing for the identification and definition of novel subspecies.

## Highlights

• A large-scale analysis of genomic data demonstrate *Salmonella* species and subspecies are genetically distinct.
• Biochemical analysis does not capture the genomic diversity of the *Salmonella* genus but routine species and subspecies identification can be achieved with rMLST
• Average Nucleotide Identify (ANI) with a 95% criteria was suitable to distinguish species and 98% to distinguish subspecies.
• Five novel *S. enteric* subspecies (VII-XI) type strains are defined.
• Reclassification of *S. arizonae* as a separate species is recommended.

## 1 Introduction

Defining prokaryotic species has long been controversial [1]. The most widely accepted approach is for organisms within a species to have (i) at least one consistent distinguishable phenotypic trait and (ii) at least 70% DNA-DNA hybridisation (DDH) [2], combining both the phenotype and genotype of isolates. Within the genus *Salmonella*, isolates are classified through a range of tests that determine biochemical properties [3]; however, conventional *Salmonella* nomenclature and taxonomy has presented problems [4,5]. Some clarity was achieved in 2005 through a judicial opinion [6] and an accompanying commentary [7], which split the genus into two species, *Salmonella bongori (S. bongori)* [6,8], (previously known as *S. enterica* subspecies V) and *Salmonella enterica (S. enterica)* [6]*.* At the time of writing, *S. enterica* was divided into six subspecies [7]: *Salmonella enterica* subspecies I (*enterica); Salmonella enterica* subspecies II (*salamae*); *Salmonella enterica* subspecies IIIa (*arizonae*); *Salmonella enterica* subspecies IIIb (*diarizonae*); *Salmonella enterica* subspecies IV (*houtenae*); and *Salmonella enterica* subspecies VI (*indica*) [7]. The phenotypic distinctions among the subspecies have been further confirmed by molecular [8,9] and genetic [5,9] tests. Prior to this opinion [6] and commentary [7], many of the *Salmonella* subspecies and serovars had at times been considered separate species [6,7] or alternatively grouped together as one subspecies including *Salmonella arizonae* and *diarizonae* [10].

A multilocus enzyme electrophoresis (MLEE) analysis [9] demonstrated the presence of a genetically distinct group, *Salmonella enterica* subspecies VII, which was later confirmed by genetic analyses [5]. As biochemical approaches identified isolates from this group as belonging to subspecies IV [9], subspecies VII has not been formally recognised [3]. A further 3 novel subspecies have also been identified, using a core genome SNPs analysis (novel A, B, and C), but at the time of writing no biochemical data had been published on these possible subspecies to ensure backward compatibility [11]. Evolutionary analysis of whole genome sequencing (WGS) data have confirmed the existence of distinct species and subspecies groups and demonstrated that extensive within-genus hybridisation has occurred during the emergence of these groups, such that their evolution cannot be modelled solely with bifurcating phylogenies [12]; however, there remains a need for reference laboratories to have a robust widely-recognised nomenclature system.

The lack of formal recognition of subspecies VII highlights the problems of a typing scheme based on biochemical characteristics [3]. It is now recognised that biochemical and microbiological subspecies identification methods are incapable of representing the genetic relationships accurately among strains [5,13]. For example, such methods require microbiological expertise across a wide range of biochemical tests and are low throughput [14]. Additionally, reference laboratories, such as Public Health England (PHE) in the UK and the US Centres for Disease Control and Prevention (CDC), have increasingly replaced biochemical approaches with whole genome sequencing (WGS), which allows greater resolution of the genetic relationships among isolates [[15], [16], [17], [18], [19], [20], [21]]. The WGS approach of core genome multilocus sequence typing (cgMLST) has been adopted as the subtyping replacement for Pulsed-field gel electrophoresis by PulseNet [22], so that global monitoring of foodborne disease can rely on this technology [22].

Whilst the move toward WGS-based subtyping allows for increased understanding and more accurate reporting, the majority of research and publications have focused on subspecies I, which is responsible for most *Salmonella* infections in humans [15,23]. However, the other subspecies are also responsible for cases of human disease [24], being opportunistic pathogens [25] that infect humans under certain conditions. Most reported cases have occurred within vulnerable individuals, such as the young [24], elderly [26], and immunocompromised [27]. Non-subspecies I *Salmonella* have been isolated from animal sources including exotic reptilian pets [28], wild [29,30] and zoo animals [31], chickens [32], livestock [33,34], and farmed reptiles [35]. The primary hosts for non-subspecies I isolates are thought to be reptiles, as isolates have been found in snake, crocodile, lizard, and turtle species [28,35,36]. Reptile farms from across the globe, including withing the United Kingdom, have had non-subspecies I salmonellae isolated from them [37].

When humans are in close contact with reptiles, the chances of transmission of non-subspecies I serovars increases [30]. In French Guiana, a correlation between *Salmonella* serotypes in patients and those found in local reptile populations was found, with two-thirds of reptile serotypes also isolated from patients [30]. A total of 16.7% of reptiles were positive for subspecies IV strains, which are responsible for ~10% of human salmonellosis cases. Reptiles are thought to be a natural reservoir for non-subspecies I organisms [36,38] and its estimated that more than 1% of households in the USA and Europe are now home to either a reptile or amphibian [31,39]. This increase in exotic pet ownership could lead to an increase in cases of salmonellosis from members of subspecies II – VI. For example, one study examining 16 pet snakes found 13 of them were infected with subspecies IIIb strains [38] and another found subspecies II strains in 8 out of 80 pet turtles, in the majority of animals more than one strain was identified [36].

The need for the characterisation of the whole of the *Salmonella* genus is accentuated by the focus on subspecies I, which skews analyses of the other subspecies [40], and by the difficulties of conventional biochemical approaches to subspecies definition and identification [13] within *Salmonella*. However, with reference laboratories adopting WGS [15,41] and increased sequencing sharing through databases [11,42], a large scale examination of the complete *Salmonella* genus is now possible. Here, a wide-ranging analysis of isolates from across the *Salmonella* genus was undertaken, with the aim of identifying and defining a genome-based approach to *Salmonella* species and subspecies definition and identification. All isolates in this study were analysed in a search for previously unidentified novel subspecies, using both genetic and biochemical approaches. Additionally, all confirmed species and subspecies as well as previously identified novel subspecies (VII, novel A, B, and C) were re-examined using statistical and phylogenetic approaches.

## 2 Materials and methods

### 2.1 Isolate collection and curation

#### 2.1.1 Non-subspecies I isolates

A total of 1530 previously published, confirmed non-subspecies I isolates [40], were used in this analysis. This dataset contained: 278 subspecies II; 308 subspecies IIIa; 442 subspecies IIIb; 326 subspecies IV; 28 subspecies IV; 22 subspecies VII;3 subspecies VIII; 48 subspecies IX; 19 subspecies X; and 56 *S. bongori* (Table S1) [40]. The subspecies of these isolates were confirmed using phenotypic data and phylogenetic clustering or reanalysis within a reference laboratory. For all of these isolates the subspecies provided for the metadata stored in Enterobase by the original submitter matched with their genetic clustering, with the exception of SAL_MA5841AA (Enterobase ID) [40].

All genomic data used in the this study is available either available in NCBI BioProject PRJNA248792 (https://www.ncbi.nlm.nih.gov/bioproject/?term=PRJNA248792), the European Nucleotide Archive (ENA Browser (ebi.ac.uk)) or Enterobase (Enterobase (warwick.ac.uk)).

#### 2.1.2 Subspecies I isolates

As subspecies I is overrepresented within the available genome databases, a representative subset was identified for analysis. This subset consisted of the twenty *S. enterica* subspecies I serovars most commonly identified in the UK at Public Health England (PHE). To assemble this dataset, eBurst Groups (eBGs), based on multilocus sequence typing (MLST) [43] were used. This approach linked identical sequence types and single-locus variants to create groups of genetically closely related isolates [43]. If a single eBG group contained isolates from only one serovar and represented more than 10% of the isolates within that serovar, then up to 10 isolates from that eBG were randomly chosen from PHE isolates. PHE isolates were used as they had been validated by a reference laboratory and they were available for reanalysis if in case of any discrepancies among the various datasets. If there were fewer than 10 isolates available from PHE, then as many as were available were used. This resulted in a subset of 271 subspecies I representative isolates (Table S1).

#### 2.1.3 Identification of novel subspecies

The 1530 non-subspecies I isolates and the 271 subspecies I isolates were used to construct rMLST [44] and cgMLST [40] minimal spanning trees (MStrees) in GrapeTree [45], indexing allelic differences. These MStrees were used to identify potential novel subspecies, defined as groups of three or more isolates that did not merge with any other subspecies when the branches in the minimum spanning trees were collapsed. Groups of three or more isolates were chosen to ensure the presence of a real subspecies and not the result of a mixed sample or a single highly mutated strain. To identify novel subspecies, branches were collapsed until the nodes of two previously identified subspecies merged or multiple phylogenetically identified subspecies merged with a confirmed subspecies, creating a single node. When a potential new subspecies was identified, Enterobase [11] was interrogated for other isolates that were closely related to these novel subspecies, which may have been uploaded without metadata or incorrect metadata and were therefore missed in the initial searches for non-subspecies I isolates. All novel subspecies were assigned the next available Roman numeral; previously detected novel subspecies A, B, and C [11] were therefore assigned subspecies VIII, IX, and X and any further detection of novel subspecies in this study were assigned XI onwards.

### 2.2 Datasets

Dataset A comprised 569 isolates representative of the diversity of *Salmonella* [6,7] and were used to reconstruct rMLST and cgMLST neighbouring joining trees [46] (NJTree), and a rMLST Maximum Likelihood (ML) phylogeny [47], and for the structure analysis of the rMLST loci. The dataset consisted of: 29 *S. bongori* isolates; 100 *S. arizonae*; 95 subspecies I; 100 subspecies II; 100 subspecies IIIb; 100 subspecies IV; 16 subspecies VI; 5 subspecies VII [9]; 3 subspecies VIII; 8 subspecies IX; 10 subspecies X; and 3 subspecies XI. Isolates were chosen from the 1801 isolates to represent the ribosomal sequence types [44] (rSTs) within each species or subspecies. Up to 100 isolates or as many as were available, all with different rSTs, were randomly chosen or as many as were available, with the exception of subspecies XI where two isolates with the same rST were used as only three isolates were available in total (Table S2).

Dataset B comprised 77 isolates and was used for cgMLST structure analysis. The 77 isolates represented seven randomly chosen examples of each suspected subspecies, except subspecies VIII, and XI, for which only three isolates were available. Isolate SAL_BA7507AA (Enterobase ID) was also included in this analysis, as it clustered with different subspecies when phylogenetic trees were reconstructed using rMLST and cgMLST sequence data. The subspecies I isolates were chosen to represent diversity, with three random examples from clades A and B [43] and 2 from the Typhi/Paratyphi A lineage [48,49], one Typhi and one Paratyphi A) (Table S2).

Dataset C comprised 25 isolates which were chosen at random for ANI comparisons. For the confirmed subspecies, type strains approved by the Judicial Commission of the International Committee on Systematics of Prokaryotes [6] were used for between species and subspecies comparisons. This included the type strain for *S. bongori, V* (NCTC12419), the type strain for *S. arizonae, IIIa* (NCTC8297) and type strains for each of the currently defined subspecies within the species *S. enterica: S. enterica* Serovar Typhimurium, I (NCTC 12416), *S. salamae*, II (NCTC5773), *S. diarizonae*, IIIb (NCTC10060), *S, houtenae*, IV (NCTC 12418) and *S. indica*, VI (NCTC12420) which had previously been defined as type strains and deposited in the NCTC culture collection. Strains of *S. enterica* subspecies VII, VIII, IX, X, and XI were also included and one of each subspecies was proposed as a type strain (Table 1).

**Table 1:** Overview of Type strains used and proposed in this study with subspecific epithets.

PubMLST_id	Reference Name	Enterobase_ID	NCTC accession number	Other collections reference^a^	Year of isolation^a^	Country of origin^a^	Source of isolation^a^	Species/subspecies No.	Species epithet	Subspecies epithet/proposed epithet
17,191	M328/NCTC20044	SAL_HA8868AA	NCTC 12416	ATCC 43971; CIP 60.62; DSM 17058; NCIMB 11450, JCM 32817	<1960	New York, USA	Unknown	I	*enterica*	*enterica* (serovar Typhimurium)
16,394	NCTC5773	SAL_HA8861AA	NCTC 5773	ATCC 43972; CIP 82.29; DSM 9220, CCUG 30039	1939	Copenhagen, Denmark	Unknown	II	*enterica*	*salamae*
16,389	NCTC10060	SAL_HA8897AA	NCTC 10060	ATCC 43973; CCUG 30040; CIP 82.31; DSM 14847; PESO M11, CCUG 30040	1947	Kentucky, USA	Unknown	IIIb	*enterica*	*diarizonae*
16,391	NCTC12418	SAL_HA8832AA	NCTC 12418	ATCC 43974;DSM 9221;CCUG 30041, CIP 82.32	<1982	Unknown	Unknown	IV	*enterica*	*houtenae*
16,393	NCTC12420	SAL_HA8831AA	NCTC 12420	ATCC 43976; CCUG 30038; CIP 102501; DSM 14848; K1240	1986	Unknown	Unknown	VI	*enterica*	*indica*
16,108	267,055	SAL_MA2040AA	NCTC 10415	*	1964	London	Unknown	VII	*enterica*	*londinensis*
16,111	85–0120	SAL_JA5200AA	NCTC 14236	*	1984	Brazil	Water	VIII	*enterica*	*brasiliensis*
15,996	304,467	SAL_MA5929AA	NCTC 14237	*	2016	Ireland	Human	IX	*enterica*	*hibernicus*
16,160	242,410	SAL_MA5883AA	NCTC 14239	*	2016	Essex	Human	X	*enterica*	*essexiensis*
16,148	267,042	SAL_MA5907AA	NCTC 10436	*	1965	South Africa	Lizard	XI	*enterica*	*reptilium*
16,406	NCTC8297	SAL_HA8865AA	NCTC 8297	ATCC 13314; CIP 8230; CN 4247; DC5; DSM 9386	<1953	Arizona, USA	Unknown	IIIa	*arizonae*	*–*
16,392	NCTC12419	SAL_HA8836AA	NCTC12419	ATCC 43975; CCUG 30042; CIP 82.33; DSM 13772	<1982	USA	Human	V	*bongori*	*–*

Table containing the type strains of the isolates used in this study. For the novel subspecies, type strains were recommended and submitted to NCTC.

a Information of previously defined strains obtained from culture collection data sheets. *also submitted to DSMZ (German Collection of Microorganisms and Cell Cultures) at time of publication

Three isolates were used for subspecies I, one isolate each from clades A and B [48] and one from the Typhi/Paratyphi A lineage [48,49], enabling an average to be taken for within subspecies I comparisons (Table S2).

Dataset D comprised 25 randomly chosen isolates used for ANI analysis. Two isolates of *S. bongori*, *S. arizonae*, subspecies II, IIIb, IV, VI, VII, VIII, IX, X, and XI respectively were chosen at random. Three isolates of subspecies I were randomly chosen, one representative each from clades A and B [48] and one from the Paratyphi A/Typhi [48,49] lineage (Table S2).

Dataset E comprised 22 representative genomes chosen from the PHE archives for a phenotypic analysis of all of the species and subspecies. Two isolates were used for each of *S. bongori, S. arizonae, S. enterica,* subspecies I, II, IIIb, IV, VI, IX, and X, with one isolate was used for subspecies VII, VIII, and XI given that only one example of these subspecies were available within PHE archives. Isolate SAL_MA5841AA, which grouped phylogenetically with subspecies I but which was typed as subspecies II using microbiological approaches, was also included for further analysis (Table S2).

### 2.3 Phylogenetic analyses

The rMLST alleles of the isolates in dataset A (Table S2) were used to reconstruct a phylogeny using the Neighbour joining algorithm [46]. The NJtree was generated using the Genome Comparator tool [42] from the Bacterial Isolate Genome Sequence database (BIGSdb) [42] available through the PubMLST website. The tree was rooted using the *S. bongori* species as an outgroup and annotations were performed using the Interactive Tree of Life (ITOL) online tool [50]. For comparison an NJtree [46] using the SalmcgMLSTv1.0 [40] was also created. In order to confirm the accuracy of the NJtree [46]the rMLST sequences were used to reconstruct a ML phylogeny [47]. The rMLST sequences of dataset A (Table S2) were exported from BIGSdb [42] after being aligned using MAFFT [51]. The ML phylongeny [47] was reconstructed using MEGA7 [52] with 100 bootstrap replications calculated using the Kimura 2-parameter model [53]. The tree was rooted using the *S. bongori* species as an outgroup and annotations were performed using the ITOL online tool [50].

### 2.4 Statistical analyses

STRUCTURE [54,55]: The structure algorithm can infer population structure accurately when compared with expected phylogenetic results [54], identifying populations from and assigning an individual to the population to which it is most similar. The algorithm analyses differences in the distribution of variants within allelic profiles of a dataset to create genetic clusters. Individuals that share similar variation patterns are grouped by a Bayesian iterative algorithm. Structure applies Markov Chain Monte Carlo (MCMC) estimations, which initially assigning individuals to a random group and then reassigns them based on estimations on variant frequency [54]. structure was applied to both rMLST [44]and cgMLST [11] [11] data, using the allele frequencies independent model, as the subspecies represented genetically diverse and distinct populations [9,11]. For both analyses, the maximum number of populations assumed (K) was increased from twelve until no further populations were observed and until lower order taxonomical groups were observed, such as the splitting of subspecies I into its respective clades [48,49].

The rMLST structure analysis was performed on dataset A (Table S2), based on 51/53 ribosomal genes. The rMLST loci BACT000060 (*rpmE*) and BACT000065 (*rpmJ*), were excluded due to the existence of paralogous loci. The rMLST analysis was run with a burn-in of 100,000 and 150,000 MCMC repetitions. The output was edited and annotated using the distruct [56] [51] software. The cgMLST analysis was based on 2746/2750 core genes. Four (STMMW_36391, STMMW_00801, STMMW_44771 and STMMW_05221) were removed from the analysis, due to the presence of paralogs in more than three of the genomes analysed. If a locus were paralogous in two or fewer genomes then the paralogous loci were replaced with the value for a missing locus. The cgMLST analysis was conducted on dataset B (Table S2), with a burn-in of 100,000 and 200,000 MCMC repetitions.

Average Nucleotide Identity (ANI) [1]: An online ANI calculator with the OrthoANIu [57] algorithm was used. This algorithm was chosen because the ANI scores between two genomes can vary when reciprocal calculations are compared [57]. Instead, OrthoANI fragments both genomic sequences and only uses the orthologous pairs to calculate the nucleotide identities. OrthoANI is highly comparable with traditional ANI but the values are typically approximately 0.1% higher [57].

The ANI values were calculated by comparing isolates from dataset C (Table S2). Pairs of isolates with ANI values higher than 95% [58,59] were considered to be from the same species. ANI analyses to identify species with the 95% criteria were performed on the entire genome sequence. As there was no percentage cut-off for subspecies, this comparison was used to identify the difference in values within and between the subspecies. The analysis was repeated using the same genomes at the levels of cgMLST and rMLST. Analysis of the WGSs of randomly chosen representatives of the subspecies (dataset D [Table S2]) were also performed to confirm the results obtained using type strains. The program OrthoANI was used to analyse isolates SAL_MA5841AA and SAL_BA7507AA against dataset C and dataset D isolates.

### 2.5 Phenotypic analyses

Metabolic phenotypes of the isolates in dataset E (Table S2), were analysed using a Biolog assay (Technopath). *Salmonella* isolates were streaked onto blood agar plates (Oxoid) and incubated at 37 °C overnight. A single colony pick was inoculated using a flat end cotton swab into type A broth (Technopath) and mixed thoroughly. Each well of a Biolog Gen III 96 -well MicroPlate, which contained metabolites or a negative control (https://biolog.com/products-portfolio-overview/microbial-identification) was inoculated using 100 μl of the broth and placed into the Biolog at an incubation temperature of 33 °C. The plate was read every 15 min for 48 h to record metabolic respiration. Runs were repeated independently 3 times.

The results were analysed by calculating the signal value [60] for each run. The signal value equalled the ([average signal over 48 h + maximum signal over 48 h]/2) – (the average signal over the first 2 h) [60]. An average signal value for each subspecies was calculated via RStudio [60] and used in order to create a heatmap of substrate utilisation for metabolic phenotyping and a dendrogram based on metabolic respiration values. The heatmap and dendrogram of metabolic phenotyping (including substrate utilisation) were generated to assess if utilisation of biochemical properties could be used to distinguish all of the *Salmonella* species and subspecies*.*

## 3 Results

### 3.1 Identification of novel subspecies

The phenotypic data for the subspecies I isolates, and phylogenetic clustering were fully congruent among the preliminary analyses as subspecies I (Fig. S1), with the exception of isolate SAL_MA5841AA, which was phenotypically identified as subspecies II, but which clustered with subspecies I isolates phylogenetically. MStrees containing all of these isolates were reconstructed using sequence data from rMLST loci (Fig. S1A) and cgMLST loci (Fig. S1b). Within the rMLST MStree, when branches were collapsed to 20/51 allelic differences (Fig. S1C), the nodes representing subspecies II and IIIb merged, whereas within the cgMLST MS tree (Fig. S1B) at 2640/2750 allelic differences subspecies VII and novel A (VIII) merged with subspecies IV (Fig. S1D). Any cluster of three or more isolates that did not cluster with any other subspecies and was present in both the rMLST and cgMLST trees was identified as a potential novel subspecies. There was one cluster of subspecies II isolates which met these criteria (isolates SAL_DA4561AA, SAL_EA2466AA and SAL_MA5907AA), these isolates were therefore identified as a novel subspecies, subspecies XI. Species *S. bongori* and *S. enterica* were distinct, as were subspecies I, II, IIIa, IIIb, IV, VI, VII, VIII, IX, X, and XI; however, subspecies IIIa was identified as a separate species, which we propose be called *Salmonella arizonae*.

### 3.2 Phylogenetic analyses

**Fig. 1:**
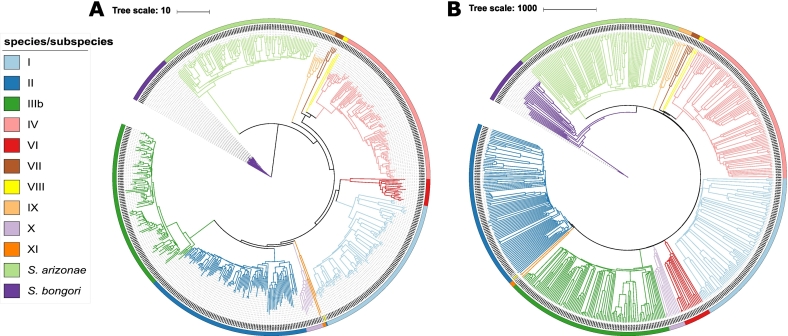
A and B: Neighbour-Joining Tree based on the rMLST loci of 569 representative isolates (A) Neighbour-Joining Tree based on the cgMLST loci of 569 representative isolates (B). Neighbour-Joining Trees of 569 isolates rooted using *S. bongori.* These trees were constructed using the BIGSdb Genome Comparator tool and SplitsTree software and edited using the ITOL online tool. Each isolate was randomly selected from the 1801 initial isolates to represent up to 100 different rST profiles per species or subspecies. A: Neighbour-Joining Tree of 569 isolates, based on rMLST. The rMLST tree clustered all of the species and subspecies into distinct clades. The only isolate that didn't cluster as expected was SAL_BA7507AA, whose subspecies name is highlighted in yellow. B: Neighbour-Joining Tree of 569 isolates, based on cgMLST. Within the cgMLST tree SAL_BA7507AA (highlighted in yellow) clustered with subspecies II but represented a sister clade to the other subspecies II isolates included in the analysis. (For interpretation of the references to colour in this figure legend, the reader is referred to the web version of this article.)

Both the rMLST (Fig. 1a) and cgMLST NJTrees [41] (Fig. 1b) of dataset A indicated that *S. bongori* was divergent from all of the other isolates, although the species did not form a single monophyletic sister clade in the cgMLST tree. *S. arizonae* (formerly *S. enterica* subspecies IIIa) was distinct from all other *S. enterica* subspecies, also forming a distinct sister clade (Fig. 1b). These trees showed that all of the *S. enterica* subspecies identified through conventional phenotypic methods (II, IIIb, IV, and VI) and those identified through phylogenetic approaches (VII [MLEE], VIII, IX, X [SNP-based MLtree], and XI [MStree]) formed distinct monophyletic groups. In both of the trees, subspecies IV, VII, and VIII formed a clade of closely related subspecies and all of the subspecies remained monophyletic. These results were highly congruent with previous analyses [5,11,61], demonstrating consistency within the identification of *Salmonella* subspecies, despite the use of different phylogenetic methods. The overall topologies of the two trees were also highly congruent, with both cgMLST and rMLST capable of subspecies discrimination. One isolate (SAL_BA7507AA) did not cluster as expected within the rMLST tree: SAL_BA7507AA was identified through phenotypic methods as a subspecies II isolate but genetically clustered with subspecies XI. Within the cgMLST tree, SAL_BA7507AA clustered with subspecies II, but formed a divergent sister clade.

In order to confirm the results observed within the NJtrees, a MLtree [47] was reconstructed for the rMLST profiles (Fig. S2). The topologies of the NJtree and the MLtree were highly congruent: *S. bongori* was divergent from all other isolates and *S. arizonae* was divergent from all the *S. enterica* subspecies. Within the rMLST MLtree, all of the subspecies nodes were highly supported with bootstrap values of 0.89 or greater.

### 3.3 Statistical analyses

**Fig. 2:**
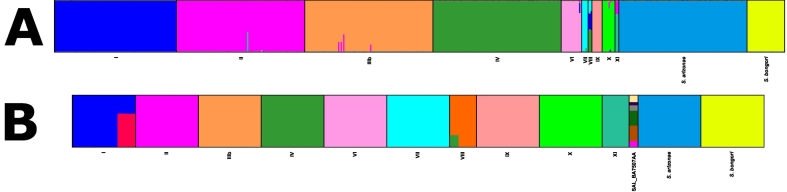
a and b: structure analysis (A) of 569 isolates using rMLST (B) of 57 isolates using cgMLST. structure analyses of representative isolates of the species and subspecies. The structure algorithm was run until no further higher-level taxonomical groups were observed. Both of the structure outputs were edited using the distruct software. A: The structure algorithm was performed on the rMLST profiles of dataset A (Table S2) isolates with a K of 12. *S. bongori*, *S. arizonae* and subspecies I, II, IIIb, IV, VI, VII, IX, and X showed very little admixture and were all distinct populations. In comparison subspecies VIII and XI were composed of multiple rMLST populations. Subspecies VIII was an admixture of subspecies VII, IV and I, all of which were present in similar proportions in each isolate. Subspecies XI was predominantly a novel population, however there was a high level of admixture from subspecies II. The isolate SAL_BA7507AA grouped with subspecies II. B: The structure algorithm was performed on the cgMLST profiles of Dataset B (Table S2) with a K of 18. Species *S. bongori* and *S. arizonae* and subspecies II, IIIb, IV, VI, VII, IX, X, and XI were all distinct populations with little admixture. Subspecies I serovars Paratyphi A and Typhi also formed a distinct population (red), but showed admixture with the rest of subspecies I. One isolate of subspecies VIII was admixed with subspecies IV. Isolate SAL_BA7507AA was highly admixed with five novel populations and some of subspecies II.

STRUCTURE: structure analyses using both rMLST and cgMLST loci (Fig. 2) also supported the distinct species status of *S. bongori*, *S. arizonae*, and *S. enterica*, along with the integrity of the *S. enterica* subspecies I, II, IIIb, IV, VI, VII, VIII, IX, X, and XI. All of the species and subspecies formed distinct and consistent clades. Structure analysis of the rMLST loci of dataset A identified twelve distinct groups (Fig. 2a). The species *S. bongori, S. arizonae* and the *S. enterica* subspecies I, II, IIIb, IV, VI, VII, VIII, IX, X, and XI were all identified as distinct populations. When the number of populations (K) was set to twelve, the species *S. bongori, S. arizonae*, and *S. enterica* subspecies I, II, IIIb, IV, VI, VII, IX, and X resolved into single populations with very little admixture. In comparison, subspecies VIII and XI were comprised of multiple rMLST populations and as such showed noticeable admixture. *S. enterica* subspecies VIII was comprised of an admixture of subspecies I, IV, and VII that were all present in similar proportions. Subspecies XI was an admixture of subspecies II and a novel population. Within the rMLST structure analysis, isolate SAL_BA7507AA clustered with the other subspecies II isolates. At an increased value of K, no further populations were observed, only increased admixture among the twelve subspecies groups was seen. The results of this structure analysis supported the presence of the twelve distinct groups, species or subspecies, and the phylogenies created from the same datasets.

By comparison, the structure algorithm performed on the cgMLST loci of dataset B isolates identified thirteen distinct groups (Fig. 2b). At a K of eighteen, structure identified species *S. bongori, S. arizonae* and the *S. enterica* subspecies I, II, IIIb, IV, VI, VII, VIII, IX, X, and XI; however, the *S. enterica* subspecies I isolates from the Typhi/Paratyphi A lineage [43,44] were admixed between the subspecies I population and a distinct population. One isolate of subspecies VIII also exhibited a small amount of admixture with subspecies IV. Using cgMLST, isolate SAL_BA7507AA also formed a distinct population that was highly admixed and was composed of five novel populations and subspecies II. Higher values of K revealed no further subspecies; subspecies became more admixed and split into lineages.

**Fig. 3:**
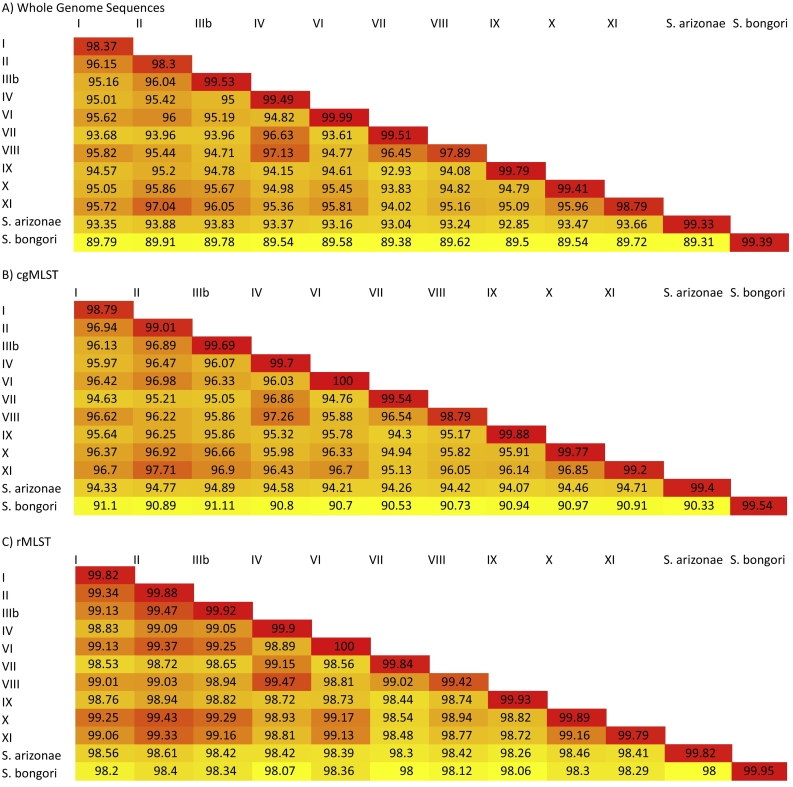
A, B and C: ANI analysis of (A) type strains and recommended type strains using WGS (B) ANI analysis of type strains and recommended type strains using cgMLSTv1.0 (C) ANI analysis of type strains and recommended type strains using rMLST. ANI analyses of isolates from dataset C (Table S2) where each isolate was compared to all of the other species or subspecies isolates and with a secondary isolate that belonged to the same species or subspecies using the OrthoANI algorithm. A: ANI analysis of type strains and recommended type strains using WGS. Within this analysis all comparisons of species and subspecies against *S. bongori* and *S. arizonae* scored below 95%. Comparisons between the other subspecies ranged between 92.93 and 97.13%, with an average of 95.15%. Comparisons within the subspecies ranged from 97.89 to 99.99%. B: ANI analysis of type strains and recommended type strains using cgMLST. *S. bongori* and *S. arizonae* scored below 95%. Comparisons between the other subspecies were between 94.3 and 97.71%, with an average of 96.10% and comparisons within subspecies were between 98.79 and 100%. C: ANI analysis of type strains and recommended type strains using the rMLST loci. The patterns observed within the rMLST analysis was very similar to that observed within the cgMLST and WGS analyses. (For interpretation of the references to colour in this figure legend, the reader is referred to the web version of this article.)

Average Nucleotide Identity (ANI): The WGS ANI performed on dataset C showed that comparisons of all of the species and subspecies against *S. bongori* scored below 90% and comparisons against *S. arizonae* scored below 95% (Fig. 3a). All of these comparisons were below the 95% cut-off typically used within ANI analyses to define species groups [58,59] and supports the reclassification of *S. enterica* subspecies IIIa into a separate species, *S. arizonae*. The presence of novel subspecies identified through phylogenetic techniques (VII, VIII, IX, X, and XI) was also supported by this ANI analysis. Comparisons among the other *S. enterica* subspecies had ANI values between 92.93% and 97.13%, with an average of 95.15%. Comparisons within the subspecies were all higher, with ANI values above 98%, except for among subspecies VIII isolates, which scored 97.89% (Fig. 3a). While this was higher than all between-species comparisons, this low score may suggest that subspecies VIII is currently comprised of multiple subspecies.

The ANI analyses were repeated using the cgMLST (Fig. 3b) and rMLST loci (Fig. 3c) and the patterns observed within these analyses were highly similar to those observed within the analysis of the WGS. When ANI was performed on the cgMLST loci *S. bongori* and *S. arizonae* were all still lower than the 95% threshold, suggesting the classification of species [58,59]. The greater relatedness within as opposed to among subspecies generally persisted with the use of rMLST loci alone, with the exception of subspecies VIII, where the within-subspecies comparison was lower in subspecies VIII than the comparison between subspecies IV and VIII (Fig. 3c).

ANI analyses on WGS, cgMLST loci, and rMLST loci were repeated using randomly chosen isolates for all of the species and subspecies (dataset D). The results were highly congruent among the chosen type strains and the randomly chosen isolates (Fig. S3). This further supported the presence of the novel subspecies (VII, VIII, IX, X, and XI) and the reclassification of *S. enterica* subspecies IIIa to *S. arizonae*. The pattern of relatedness within ANI analyses using WGS, cgMLST, and rMLST sequence data were consistent throughout the comparisons, demonstrating that subspecies are observable across different genomic levels, despite a marked reduction in the number of loci used per analysis.

Isolate SAL_BA7507AA, which clustered with subspecies XI in rMLST trees and with subspecies II using cgMLST, was analysed using ANI against both datasets C and D. This isolate was closely related to subspecies II and XI, scoring 97.99% and 97.87% respectively for dataset C and 97.65% and 97.69% respectively for dataset D (Fig. S4a). These values were all lower than comparisons within the subspecies II and subspecies XI isolates, which all scored above 98%. Whereas when isolate SAL_MA5841AA, which was phenotypically typed as subspecies II but clustered with subspecies I isolates phylogenetically, was analysed against datasets C and D it was very closely related to subspecies I, scoring 99.89% and 99.98% respectively (Fig. S4b).

### 3.4 Phenotypic analyses

**Fig. 4:**
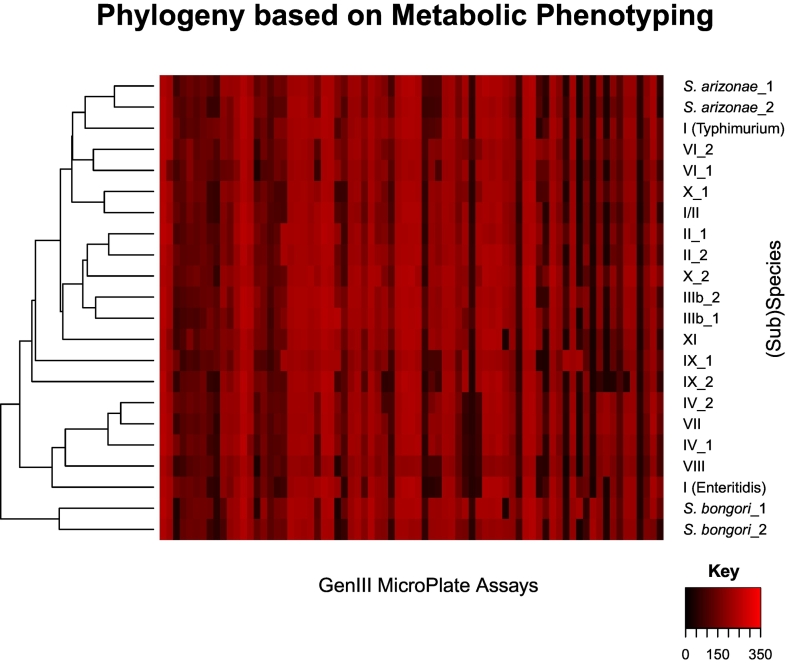
Dendogram based on metabolic phenotyping. Pairs of isolates from each (sub)species underwent metabolic phenotyping using the biolog GenIII MicroPlate Assays. Runs were conducted three times and an average of the three runs was used to create signal values. These values were used to construct a dendrogram based on metabolic respiration values.

Metabolic phenotyping was performed on pairs of isolates from each species and subspecies and the average signal values [60] used to construct a dendrogram based on metabolic respiration values (Fig. 4). This indicated that both *S. bongori* and *S. arizonae* (currently recognised subspecies IIIa) isolates clustered as species. This clustering was the case for the conventionally defined subspecies II, IIIb, IV, and VI and the phenotypically identified subspecies XI also formed an independent branch. The signal values clustered subspecies VII [9] and VIII [11] with subspecies IV. Subspecies I and X isolates did not cluster with each other or other subspecies (Fig. 4). The signal values identified subspecies I, serovar Enteritidis as phenotypically most closely related to the subspecies IV, VII, and VIII clade, while serovar Typhimurium was phenotypically most closely related to the *S. arizonae* clade. One of the subspecies X isolates was divergent from to the subspecies II isolates while the other clustered with isolate I/II (SAL_MA5841AA). The biochemical approaches and the PHE k-mer approach identified this isolate as subspecies II, but it had clustered phylogenetically with subspecies I isolates. The subspecies IX isolates did not form a single clade (Fig. 4) but were both divergent from the same clade. Prior to identification and subsequent reclassification as novel subspecies, subspecies IX, X, and XI were all identified as subspecies II with phenotypic data accompanying the isolate; however, the dendrogram produced from the signal values demonstrated that none of these subspecies were in the same clade as subspecies II (Fig. 4). In addition, subspecies IX and XI were not closely related phenotypically to subspecies II.

Proposal of type strains and epithets for novel subspecies

The type strains for subspecies VII, VIII, IX, X, and XI (Table 1) recommended here were submitted to the National Collection of Type Cultures (NCTC) database and have therefore been given NCTC accession numbers as follows: Subspecies VII, SAL_MA2040AA, NCTC 1041; subspecies VIII, SAL_JA5200AA, NCTC 14236; subspecies IX, SAL_MA5929AA & SAL_MA5932AA, NCTC 14237 & NCTC 14238 respectively; subspecies X, SAL_MA5883AA & SAL_MA5962AA, NCTC 14239, NCTC 14240 respectively; and subspecies XI, SAL_MA5907AA, NCTC 10436. Strains were also submitted to the German Collection of Microorganisms and Cell Cultures (DSMZ) at time of publication and currently awaiting DSMZ accession numbers.

Type strains and epithets proposed in this study for recently defined subspecies included *S. londinensis*, VII (NCTC10415), *S. brasiliensis*, VIII (NCTC14236), *S. hibernicus*, IX (NCTC14237), *S. essexiensis*, X (NCTC14239) and *S. reptilium* XI (NCTC10436) (Table 1). Naming of the subspecies was undertaken in line with the International Code of Nomenclature of Prokaryotes [62,63].

## 4 Discussion

The availability of very large numbers of bacterial WGSs provides alternative approaches to the definition and identification of bacterial species and subspecies. At the time of writing, isolate characterisation of *Salmonella* remained primarily undertaken by morphological and metabolic characterisation, despite the widespread uptake of WGS by reference laboratories, including those of Public Health England (PHE) isolates [[15], [16], [17], [18], [19], [20],^41^] and the U.S. Centers for Disease Control and Prevention (CDC) [21,64,65]. The phenotypic approaches were prone to result in misclassifications [40] with a lack of clarity in the identification of species and subspecies, as biochemical properties were often shared among distinct organisms [5,13]. Furthermore, the adoption by PulseNet International of cgMLST [22,65], a WGS-based method, for the global monitoring of foodborne disease outbreaks [15] and analysis [22] including for *Salmonella*, necessitated the harmonisation of approaches to species and subspecies definition and identification. WGS data have the additional advantage of being suitable for evolutionary analysis [12].

Using WGS-based approaches, four new *S. enterica* subspecies were confirmed and named with the next available roman numerals, in accordance with current taxonomy [4]: subspecies VII [9]; VIII; IX; and X. An additional novel subspecies was also identified, subspecies XI. Furthermore, *S. enterica* subspecies IIIa was confirmed as a separate species, *Salmonella arizonae*. The name *S. arizonae* was chosen as *arizonae* was the historical name for subspecies IIIa before the subspecies were reclassified with roman numerals [4]. Until 1973, isolates from this group were considered to form a separate species [10]. The identification, characterisation, and reclassification of these *Salmonella* species and subspecies was consistent across different genomic levels, using multiple phylogenetic and statistical approaches [54,57] which were highly congruent. As different subspecies and serovars of *Salmonella* have different antimicrobial resistance profiles [66,67], virulence properties, and primary hosts [66,67], the identification and accurate analyses of these different subspecies, such as with the cgMLST genus scheme (SalmcgMLSTv1.0) [40], is important.

WGS-based methods consistently identified the subspecies correctly and within the phylogenies the species and subspecies formed distinct clades irrespective of the phylogenetic approach used. Trees reconstructed using the Neighbour-Joining algorithm, based on the 51 rMLST loci [44] and the 2750 cgMLST [40] loci (Fig. 1), and a rMLST phylogeny reconstructed using the Maximum Likelihood algorithm [46] (Fig. S2) were highly congruent. The consistencies between the MLtree and NJtree demonstrated that the increase in time taken to produce a higher resolution tree provided limited further insight into the relationships among the salmonellae and was therefore unnecessary for the purpose of species and subspecies classification. The phylogenetic clades were also highly congruent with the results from ANI [57] (Fig. 3) and structure [54] analyses (Fig. 2), where all species and subspecies were identified. All these analyses supported the classification of *S. bongori, S. arizonae* and *S. enterica* as separate species and of the division of *S. enterica* into subspecies I – XI. In addition, these groupings were consistent with those defined using alternative evolutionary approaches, which in addition showed evidence for hybridisation among the species and subspecies during their emergence [12].

In all the reconstructed phylogenies, *S. bongori* was the most divergent of the *Salmonella* species. Furthermore, *S. arizonae* (currently recognised as subspecies IIIa) was divergent from the remaining *S. enterica* subspecies in all phylogenies, consistent with it forming another species and being a *S. enterica* subspecies. Results from the ANI [57] analyses also demonstrated that both *S. bongori* and *S. arizonae* were separate species, as defined by the 95% ANI threshold for species definition. Comparisons with *Salmonella* isolates from the other species and subspecies consistently scored below 95% ANI [1], *S. bongori* consistently scored below 90% when compared against all other *Salmonella* species or subspecies and *S. arizonae* scored below 94%.

The genetic differences among the subspecies were also conserved, as were the relationships among the species and subspecies, observable within phylogenies when trees were constructed using cgMLST [40] or rMLST [44], within ANI analyses and using the structure algorithm. The ANI values were consistently lower among subspecies comparisons than within subspecies comparisons. When using structure, the rMLST analysis identified all twelve of the species and subspecies at a population of twelve, while cgMLST required a population of eighteen. As anticipated, in the cgMLST analysis, serovars Typhi and Paratyphi A were split into their own population, which was admixed with the subspecies I population. It is likely this distinct population occurred due to the host-restricted nature of these serovars [68,69], the reduction in their genome size [70], and hybridisation between the two serovars [69,70]. Furthermore, one isolate of subspecies VIII also demonstrated a degree of admixture with subspecies IV, this is likely because these subspecies are closely related [11].

Of the large number of isolates examined here, only two isolates were identified that demonstrated incongruences. When analysed using phylogenetic approaches isolate SAL_BA7507AA clustered with subspecies XI using rMLST [44] and subspecies II using cgMLST [40]. Isolate SAL_BA7507AA was also problematic when the cgMLST loci were analysed using the structure algorithm [54] (Fig. 2b), showing evidence of being highly admixed. ANI analysis of this isolate (Fig. S4a) against the type strains (Table 1) and randomly chosen isolates (Datasets C and D) found that it was on the threshold for clustering with both subspecies II (97.65% and 97.99% respectively) and XI (97.69% and 97.87% respectively). The ambiguity to which subspecies isolate SAL_BA7507AA belongs in all these analyses suggests that it could represent a novel subspecies; however, this isolate could also be an admixed sample or an isolate representing a midpoint between subspecies II and XI. Additional isolates closely related to SAL_BA7507AA would need to be identified to confirm the classification of this isolate.

All of the WGS-based approaches used in the present study identified isolate SAL_MA5841AA as a subspecies I organism, despite it being identified as a subspecies II using conventional approaches. Reanalysis using Biolog (Technopath) indicated that this isolate clustered more closely with isolates identified as subspecies II. This discrepancy suggested that occasional conflicts between biochemical and phylogenetic approaches will occur, but that these appear to be rare. This further emphasised the need for a WGS-based approach to species definition and identification, demonstrating the misclassifications [40] that can occur with phenotypic biochemical properties, which are frequently shared [5,13]. Furthermore, dendrograms produced from the metabolic respiration values demonstrated that phenotypic typing is not always capable of delineating the *Salmonella* subspecies. The large-scale analyses carried out on a selection of 22 *Salmonella* isolates using WGS-based approaches are not scalable to phenotypic approaches. These 22 isolates were representative of all of the *Salmonella* species and subspecies identified through both conventional and phylogenetic approaches. The metabolic respiration [60] of these isolates was examined using Biolog (Technopath) (Fig. 4). While some of the *Salmonella* species and subspecies were identified through these metabolic respiration profiles, the dendrogram produced demonstrated that phenotypic typing was not consistent in distinguishing *Salmonella* subspecies. This shortcoming was true for both conventionally-identified subspecies, such as subspecies I and for phylogenetically identified subspecies such as IX and X [11]. The representative isolates of these subspecies did not form subspecies-specific clades within the dendrogram of the metabolic respiration values. The isolates from phylogenetically identified subspecies VII [9] and VIII [11] clustered with the subspecies IV isolates, which was expected, as subspecies VII and VIII isolates were classified as subspecies IV prior to the present analyses. While there were phenotypic differences between most of the subspecies, shared biochemical properties [5,13] resulted in the inability for biochemical subspecies identification methods to represent the genetic relationships among isolates accurately [5,13] and consistently distinguish all of the subspecies, demonstrating the need for a shift in how subspecies are identified [1]. Furthermore, these methods require microbiological expertise across a wide range of biochemical tests and are low throughput [14].

A number of studies have been published which identify the need for a genome-based species identification methods [[71], [72], [73]] and recommended guidelines have been published [72,74,75] in support of this shift away from conventional methods. The use of genome-based methods for the identification of species is becoming more common and species within other genera, such as *Neisseria* [76] and *Acinetobacter* [77] have been identified using WGS-based approaches. In some cases, with open sharing of genomic data, similar studies have overlapped [11,12]. Even though genomic approaches were different, the same novel groups have been detected but named in different ways, for example novel subspecies VIII (novel subsp. A, Houtenae B), IX (novel subsp. B, VIII) and X (novel subsp. C, Salamae B)) [11,12]. Hence the need for the IJSEM, which ultimately agrees new taxonomy proposals and has now introduced the use of genomic data for publication of new taxa into their scope (www.microbiologyresearch.org/journal/ijsem/scope). Having bespoke genomic methods can be insightful in understanding evolutionary events and detection of novel strains but what standardised methods can be used in reference laboratories to detect and report out these strains in real-time? Routinely, *Salmonella* isolates can be typed using sequence typing methods, such as cgMLST [78] or rMLST [44] and isolates that do not cluster with any known subspecies can be confirmed as novel subspecies using ANI [57], the new ‘gold standard approach’ [59] for species definition and identification. Of course, updated k-mer databases can be used and should be reviewed to add any newly defined species or subspecies. As The cgMLST phylogenies provided limited additional resolution compared to the rMLST phylogenies, and given the facts that rMLST requires less computational power and curation of fewer loci [44,79], rMLST could be used for routine species and subspecies identification. The ribosomal genes are distributed around the genome, offering some stability against horizonal gene transfer [44,79] and are also under stabilising selection for conservation of function [44]. These traits make the ribosomal genes ideal candidates for a typing scheme.

Isolates from the different *S. enterica* subspecies began to cluster together when more than 19 of the rST alleles differed. Therefore, a more conservative number of allelic differences, such as 15, would identify the majority of isolates from the same subspecies, whilst ensuring that conflicts among subspecies classification did not occur. This threshold should, however, be periodically reviewed because as gaps within the diversity of an organism are filled, the number of allelic differences required to identify all members of a species or subspecies can decrease.

For the confirmation of novel subspecies, ANI can be used by comparing test WGS data against reference genomes for each subspecies. To identify organisms as the same species an ANI of 95% or greater is generally recommended, although this varies among species [58], from the data presented here, 95% appears appropriate for the salmonellae. ANI has been shown to be comparable [59] and congruent [80] to DNA-DNA hybridisation (DDH) [2]. Due to the development of online tools [57,81], however, ANI analyses are simpler to perform than DDH, which is too labour-intensive [72], time consuming [72] and error prone [82] for all but a few labs to perform [72,82]. As ANI indexes differs from across the whole genome, it is not reliant on a single or small subset of genes remaining consistent across all isolates within a species or subspecies. Therefore, ANI should not be affected by small changes within a genome and should be capable of consistently identifying species and subspecies.

Five new *S. enterica* subspecies have been confirmed and named with the next available roman numerals, in accordance with current Salmonella taxonomy (VII, VIII, IX, X, and XI) [3,4] with the subspecific epithet in line with the International Code of Nomenclature of Prokaryotes [62,63] (Table 1). Representative isolates have been deposited into the NCTC and DSMZ culture collections (Table 1) as type strains (*S. enterica* subsp. *londinensis*, VII: NCTC 1041, *S. enterica* subsp. *brasiliensis*,VIII: NCTC 14236, *S. enterica* subsp. *hibernicus* IX: NCTC 14238 and NCTC 14237,*S. enterica* subsp. *essexiensis*, X: NCTC 14239 and NCTC 14240, *S. enterica* subsp. *reptilium,* XI: NCTC 10436). *S. enterica* subspecies IIIa was identified as a separate species – *Salmonella arizonae*, as historically suggested [83] which was identified in this study using rMLST and cgMLST NJtrees and confirmed using an MLTree, structure analysis and ANI data. It is demonstrated here that the identification of species and subspecies of the genus *Salmonella* can and should be performed using WGS-based approaches. This methodology change would be in line with global surveillance [22] and reference laboratories [15], which are now routinely using WGS-based approaches for *Salmonella* identification and monitoring.

## 5 Conclusions

A comprehensive analysis of the genus of *Salmonella* with respect to species and subspecies structure, undertaken with WGS data analysed with a range of different approaches, proposed that: (i) five novel subspecies (*S. enterica* subsp. *londinensis* (VII), *S. enterica* subsp. *brasiliensis* (VIII), *S. enterica* subsp. *hibernicus* (IX), *S. enterica* subsp. *essexiensis* (X), *S. enterica* subsp. *reptilium* (XI)) be defined; and (ii) *S. enterica* subspecies IIIa should be reclassified as the separate species, *S. arizonae.* The analyses demonstrated that conventional microbiological approaches to species and subspecies definition and identification were not uniformly consistent with WGS data and could not identify all of the species and subspecies within the genus reliably. It was further shown that routine species and subspecies identification could be achieved with rMLST, which it has a low computational overhead. For those few isolates which were not identified unambiguously with rMLST data alone, ANI using core genome loci confirmed species or subspecies status is required.

The following are the supplementary data related to this article.Supplementary Fig. 1 a, b, c and d: Minimal Spanning Trees calculated using GrapeTree of all isolates (A) calculated using rMLST (B) calculated using cgMLST (C) calculated using rMLST and collapsed to 20/51 loci different and (D) calculated using cgMLST and collapsed to 2640/2750 loci different.
Minimal Spanning Trees created using the GrapeTree algorithm, using all 1801 isolates. This dataset is composed of 271 subspecies I, 278 II, 308 IIIa, 442 IIIb, 326 IV, 28 VI, 22 VII, 3 Novel A, 48 Novel B, 19 Novel C, and 56 *S. bongori* and is labelled according to the original metadata. These isolates cover all non-subspecies isolates and a subset of the most common *S. enterica* subspecies I isolates.
A: Minimal Spanning Tree created using GrapeTree on the 51 rMLST loci. Isolate SAL_MA5841AA (node circled in blue), was the only isolate that did not cluster as expected.
B: Minimal Spanning Tree created using GrapeTree on the 2750 cgMLST loci. Isolate SAL_MA5841AA (node circled in blue), was the only isolate that did not cluster as expected.
C: Minimal Spanning Tree created using GrapeTree on the rMLST loci and collapsed to differences of 20/51 loci, at this point the major subspecies II grouping merged with the subspecies I isolates. Isolates that did not cluster with any other subspecies but formed a cluster of three or more isolates were ideal candidates for novel subspecies. This led to the identification of novel subspecies XI (red circle). The other isolates that did not cluster with subspecies II were not of three or more isolates, however searches in Enterobase for further isolates closely related to them were conducted and no similar isolate were found.
D: Minimal Spanning Tree created using GrapeTree on the cgMLST loci and collapsed to differences of 2640/2750 loci, at this point both the subspecies VII and Novel A (VIII) subspecies groups merged with the subspecies IV isolates. Isolates that did not cluster with any other subspecies but formed a cluster of multiple isolates were ideal candidates for novel subspecies. This led to the identification of novel subspecies XI (red circle). Supplementary Fig. 1

Supplementary Fig. 2 A maximum-likelihood tree based on the rMLST loci of 569 representative isolates.
Maximum-Likelihood tree of dataset A isolates. Using MAFFT rMLST profiles were aligned and exported from BIGSdb, these profiles were then used to create a Maximum-Likelihood tree using muscle with 100 bootstrap replications using the Kimura 2-parameter model [48]. All species and subspecies groups were supported by a bootstrap of over 0.8 (0.82–1.00). Isolate SAL_BA7507AA (highlighted in yellow) clustered with subspecies XI. Supplementary Fig. 2

Supplementary Fig. 3 a, b and c: ANI analysis of randomly selected isolates of each species and subspecies (A) WGS (B) ANI) cgMLST (C) rMLST.
ANI analyses of randomly selected isolates for each species and subspecies. Each isolate was compared to an isolate of all the other species or subspecies and with a secondary isolate that belonged to the same species or subspecies using the OrthoANI algorithm.
A: ANI analysis of random isolates using WGS. All comparisons of species and subspecies against *S. bongori* and *S. arizonae* scored below 95%. Comparisons between the other subspecies ranged between 93.14 and 97.55%. Comparisons within the subspecies ranged from 98.41 to 99.94%. These results were highly congruent with the results from the type strain analysis.
B: ANI analysis of random isolates using cgMLST. The cgMLST analysis was congruent with the WGS ANI results and with the cgMLST analysis of type strains. *S. bongori* and *S. arizonae* scored below 95%. Comparisons between the other subspecies scored between 94.32 and 98.11% and comparisons within subspecies scored between 99.8 and 99.96%.
C: ANI analysis of random isolates using the rMLST loci. The patterns observed within the rMLST analysis were congruent with the cgMLST and WGS analyses and the results observed using the type strains. Supplementary Fig. 3

Supplementary Fig. 4 a and b: ANI analysis using both randomly selected representatives of subspecies and type strain of (A) isolate SAL_BA7507AA and (B) isolate SAL_MA5841AA.
ANI analyses of the isolates within this study which did not cluster consistently. These isolates were compared to representatives of the other subspecies. The results are presented in a heatmap from red – yellow, representing most to least related.
A: ANI analysis comparing the WGS of SAL_BA7507AA with the WGS of representative isolates for each other species and subspecies. The values for SAL_BA7507AA when compared to subspecies II and XI were coloured red, indicating close relatedness.
B: ANI analysis comparing the WGS of SAL_MA5841AA with the WGS of representative isolates for each other species and subspecies. Values for SAL_MA5841AA when compared to subspecies I scored in red. Supplementary Fig. 4

Supplementary Table 1 Table of all isolates within the MSTree analyses.
A table of all of the isolates used in the MSTree analyses, including isolate names for both PubMLST and Enterobase as all isolates are available within both databases, enabling users to look up all isolates in either database. The subspecies provided by the isolate metadata and that used within the analysis are also shown. Supplementary Table 1

Supplementary Table 2 Table of all datasets used for each analysis.
A table of all of the isolates that were used in analyses which involved a subset of all of the isolates and which dataset they belong. Both pubMLST and Enterobase Ids are provided for users to be able to locate isolates in either database. Analysis subspecies is also indicated. Supplementary Table 2

## Declaration of competing interest

None.
