# Supplementary figures and images for: An evaluation of the species and subspecies of the genus Salmonella with whole genome sequence data: Proposal of type strains and epithets for novel S. enterica subspecies VII, VIII, IX, X and XI
Source: Genomics. 2021 Sep;113(5):3152–62. doi: 10.1016/j.ygeno.2021.07.003 (PMC8426187; doi:10.1016/j.ygeno.2021.07.003)

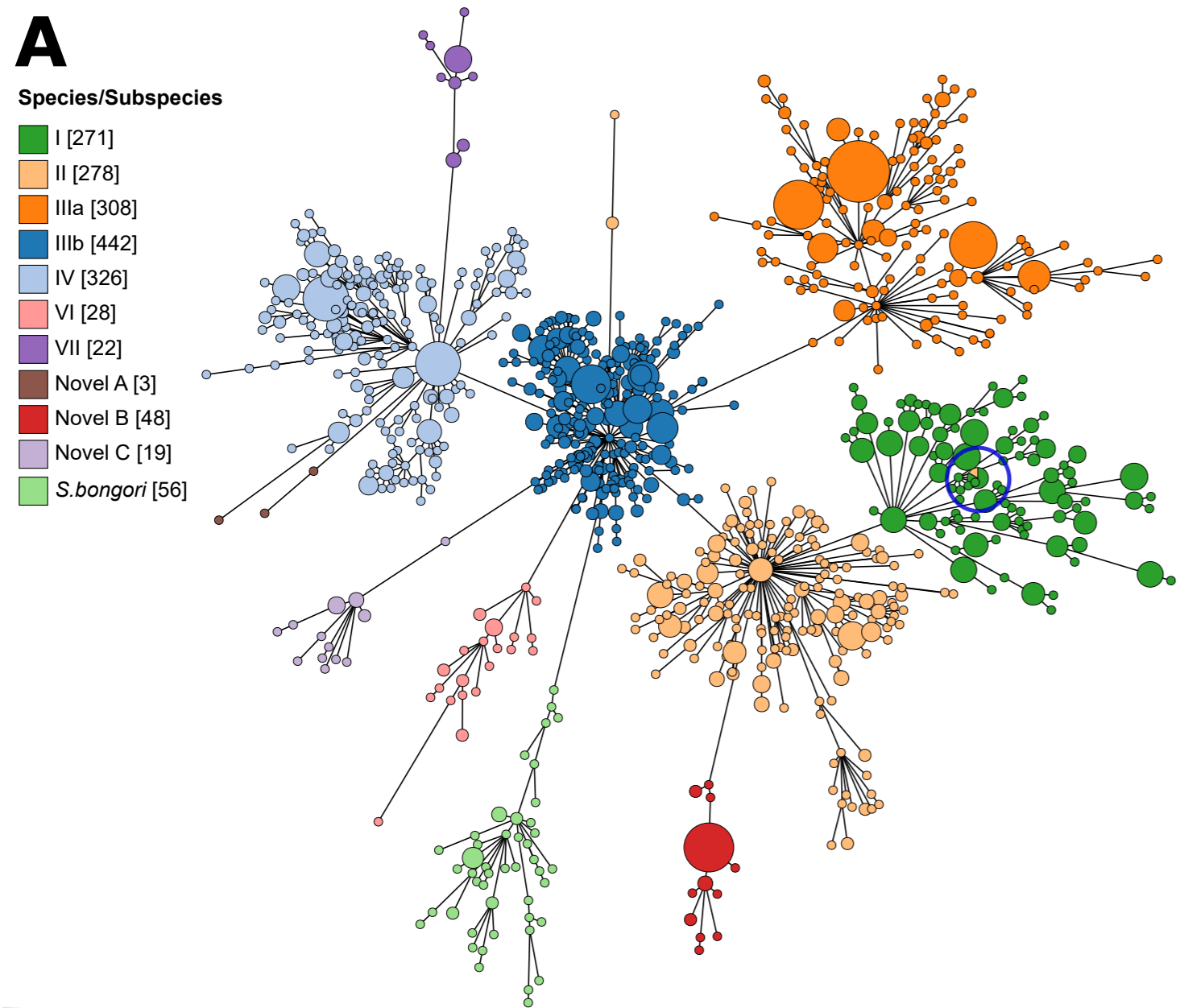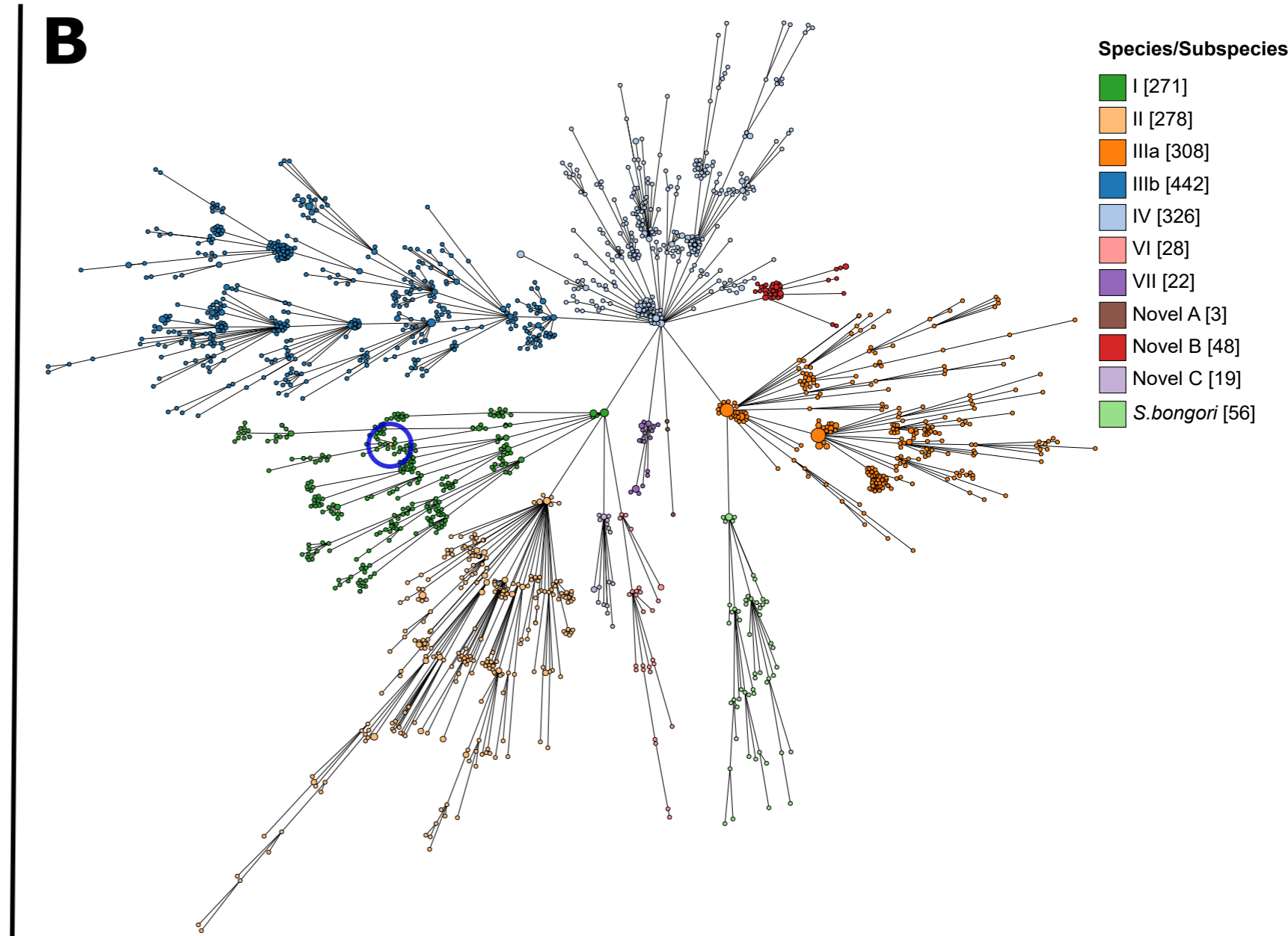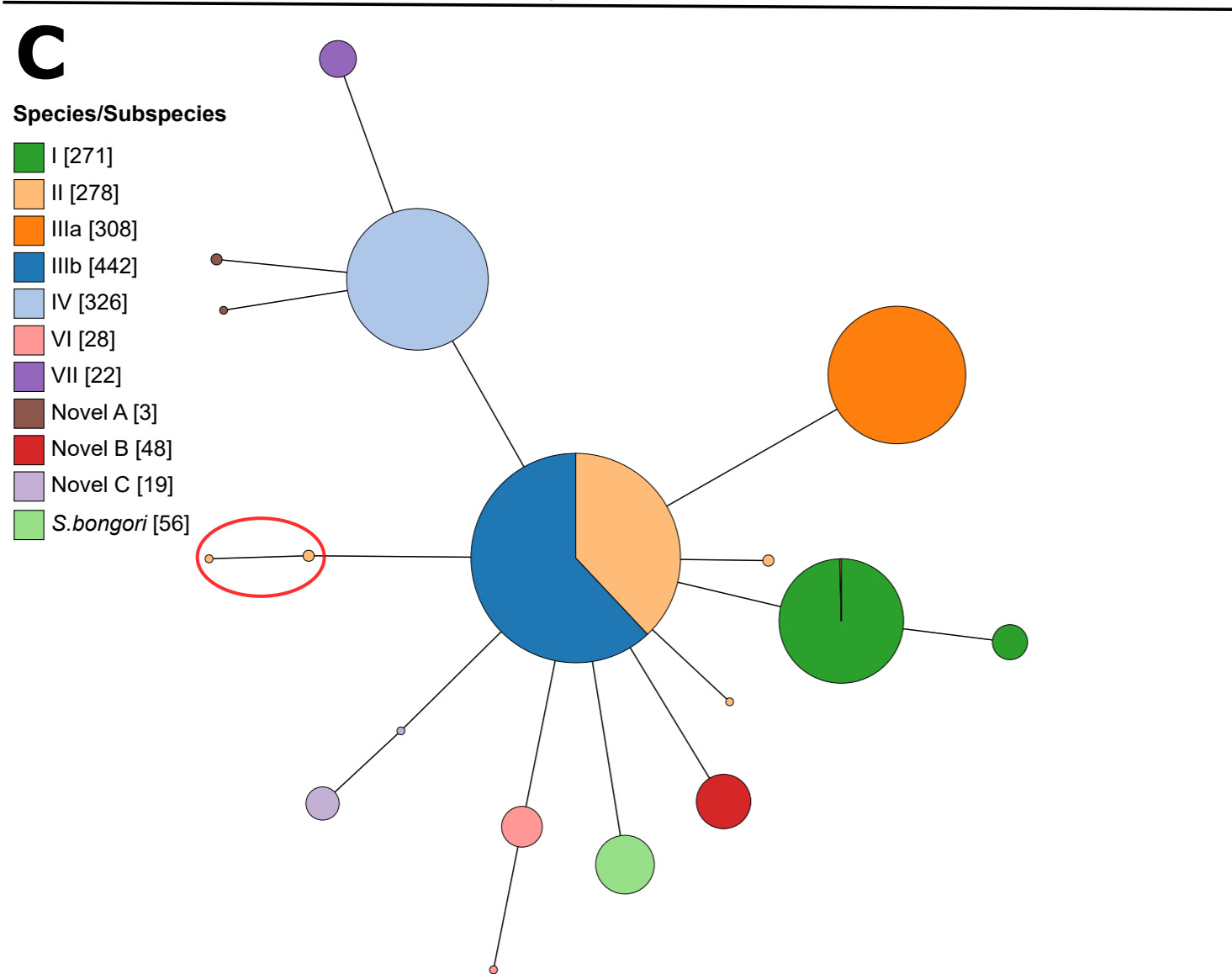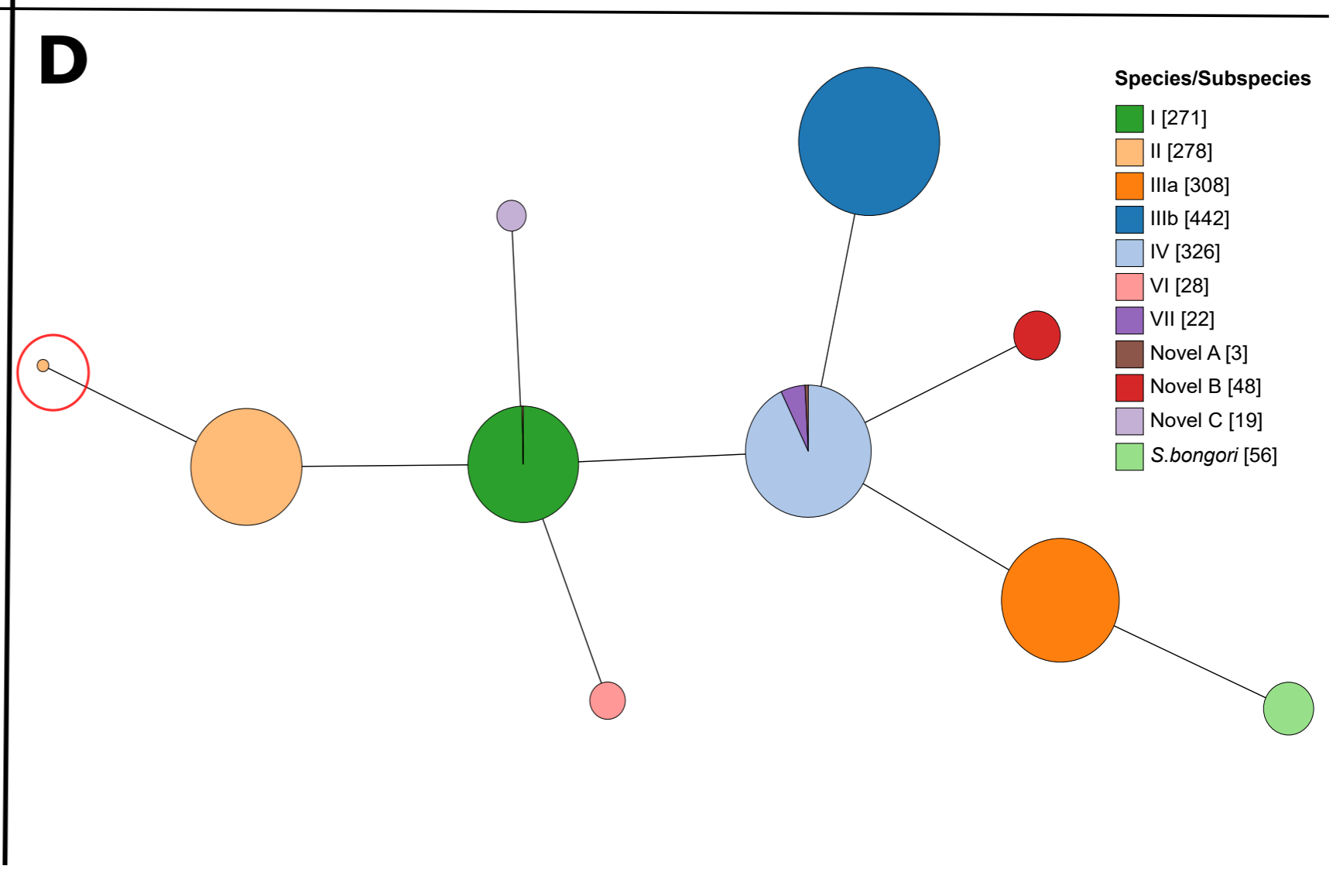

Supplement: The following are the supplementary data related to this article.Supplementary Fig. 1 — a, b, c and d: Minimal Spanning Trees calculated using GrapeTree of all isolates (A) calculated using rMLST (B) calculated using cgMLST (C) calculated using rMLST and collapsed to 20/51 loci different and (D) calculated using cgMLST and collapsed to 2640/2750 loci different. Minimal Spanning Trees created using the GrapeTree algorithm, using all 1801 isolates. This dataset is composed of 271 subspecies I, 278 II, 308 IIIa, 442 IIIb, 326 IV, 28 VI, 22 VII, 3 Novel A, 48 Novel B, 19 Novel C, and 56 S. bongori and is labelled according to the original metadata. These isolates cover all non-subspecies isolates and a subset of the most common S. enterica subspecies I isolates. A: Minimal Spanning Tree created using GrapeTree on the 51 rMLST loci. Isolate SAL_MA5841AA (node circled in blue), was the only isolate that did not cluster as expected. B: Minimal Spanning Tree created using GrapeTree on the 2750 cgMLST loci. Isolate SAL_MA5841AA (node circled in blue), was the only isolate that did not cluster as expected. C: Minimal Spanning Tree created using GrapeTree on the rMLST loci and collapsed to differences of 20/51 loci, at this point the major subspecies II grouping merged with the subspecies I isolates. Isolates that did not cluster with any other subspecies but formed a cluster of three or more isolates were ideal candidates for novel subspecies. This led to the identification of novel subspecies XI (red circle). The other isolates that did not cluster with subspecies II were not of three or more isolates, however searches in Enterobase for further isolates closely related to them were conducted and no similar isolate were found. D: Minimal Spanning Tree created using GrapeTree on the cgMLST loci and collapsed to differences of 2640/2750 loci, at this point both the subspecies VII and Novel A (VIII) subspecies groups merged with the subspecies IV isolates. Isolates that did not cluster with any other subspecies but formed a cluster of multiple isolates were ideal c [file mmc1.pdf]

Tree scale: 0.001

## species/subspecies

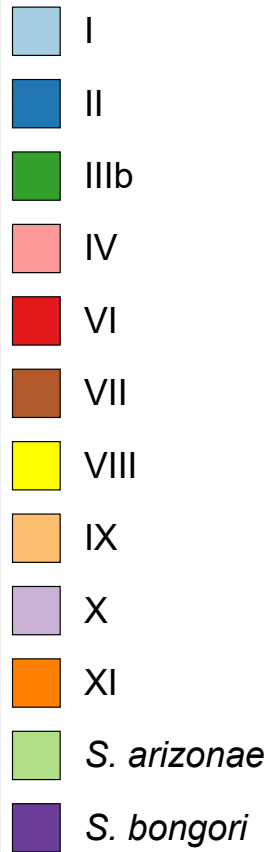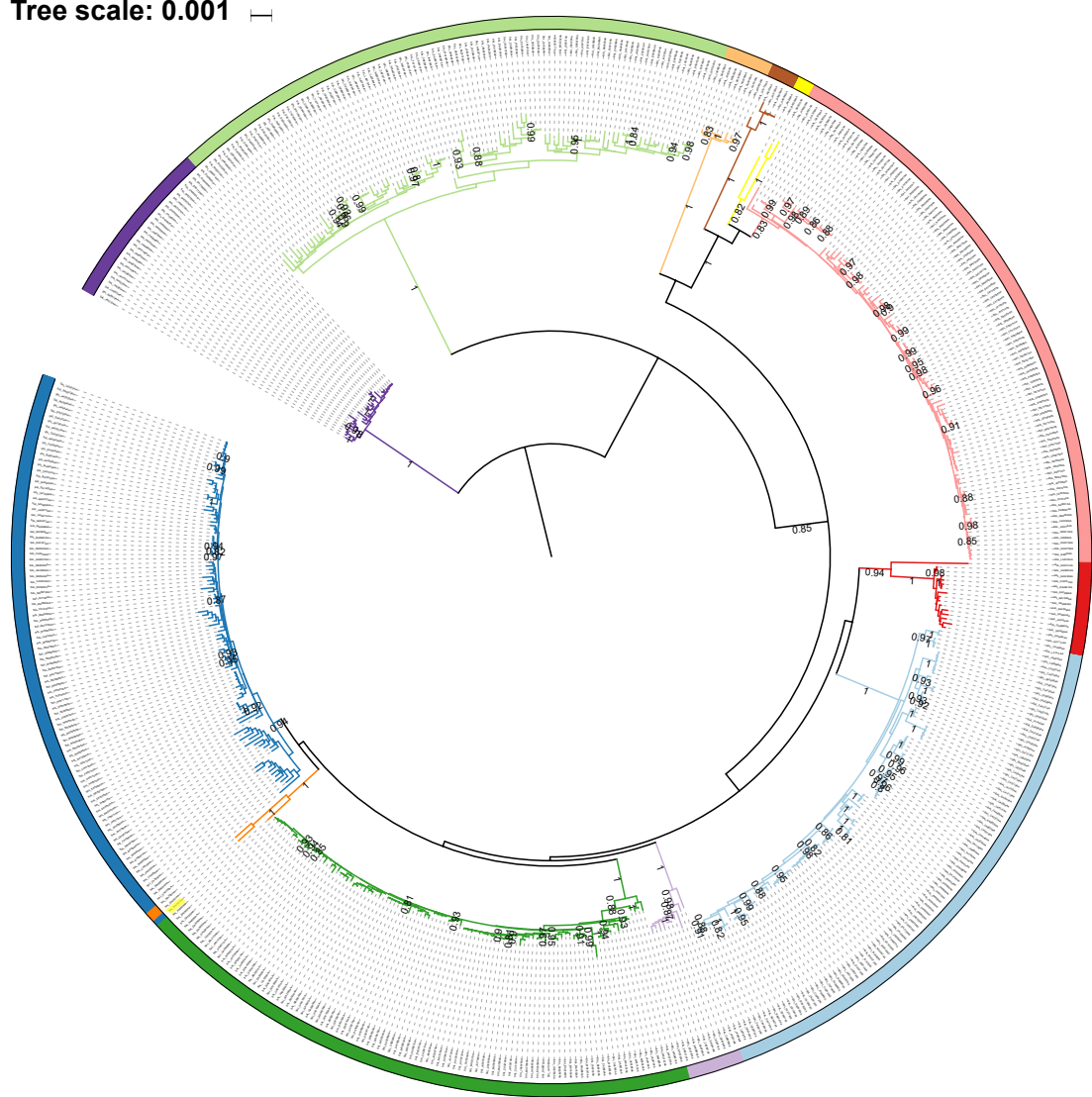

Supplement: Supplementary Fig. 2 — A maximum-likelihood tree based on the rMLST loci of 569 representative isolates. Maximum-Likelihood tree of dataset A isolates. Using MAFFT rMLST profiles were aligned and exported from BIGSdb, these profiles were then used to create a Maximum-Likelihood tree using muscle with 100 bootstrap replications using the Kimura 2-parameter model [48]. All species and subspecies groups were supported by a bootstrap of over 0.8 (0.82–1.00). Isolate SAL_BA7507AA (highlighted in yellow) clustered with subspecies XI. [file mmc2.pdf]
