# Supplementary material for: An evaluation of the species and subspecies of the genus Salmonella with whole genome sequence data: Proposal of type strains and epithets for novel S. enterica subspecies VII, VIII, IX, X and XI
Source: Genomics. 2021 Sep;113(5):3152–62. doi: 10.1016/j.ygeno.2021.07.003 (PMC8426187; doi:10.1016/j.ygeno.2021.07.003)
Supplement: Supplementary Fig. 3 — a, b and c: ANI analysis of randomly selected isolates of each species and subspecies (A) WGS (B) ANI) cgMLST (C) rMLST. ANI analyses of randomly selected isolates for each species and subspecies. Each isolate was compared to an isolate of all the other species or subspecies and with a secondary isolate that belonged to the same species or subspecies using the OrthoANI algorithm. A: ANI analysis of random isolates using WGS. All comparisons of species and subspecies against S. bongori and S. arizonae scored below 95%. Comparisons between the other subspecies ranged between 93.14 and 97.55%. Comparisons within the subspecies ranged from 98.41 to 99.94%. These results were highly congruent with the results from the type strain analysis. B: ANI analysis of random isolates using cgMLST. The cgMLST analysis was congruent with the WGS ANI results and with the cgMLST analysis of type strains. S. bongori and S. arizonae scored below 95%. Comparisons between the other subspecies scored between 94.32 and 98.11% and comparisons within subspecies scored between 99.8 and 99.96%. C: ANI analysis of random isolates using the rMLST loci. The patterns observed within the rMLST analysis were congruent with the cgMLST and WGS analyses and the results observed using the type strains. [file mmc3.pdf]

A) Whole Genome

	I	II	IIIb	IV	VI	VII	VIII	IX	X	XI	S. arizonae	S. bongori
I	98.41											
II	95.98	99.28										
IIIb	94.95	96.12	99.48									
IV	94.96	95.67	94.75	99.32								
VI	95.63	96.08	95.27	94.78	99.51							
VII	93.66	94.07	93.83	96.61	93.58	99.5						
VIII	95.94	95.58	94.83	97.15	94.85	96.27	99.94					
IX	94.65	95.26	94.87	94.05	94.67	93.14	94.23	99.73				
X	95.29	96.16	95.67	94.83	95.33	93.68	94.74	94.82	98.91			
XI	95.78	97.55	95.96	95.28	95.85	94.03	95.23	95.09	95.94	98.79		
S. arizonae	93.47	93.76	93.82	93.26	93.1	92.94	93.31	92.92	93.43	93.61	99.47	
S. bongori	89.96	89.79	89.85	89.45	89.57	89.35	89.59	89.68	89.6	89.98	88.99	99.3

B) cgMLST

	I	II	IIIb	IV	VI	VII	VIII	IX	X	XI	S. arizonae	S. bongori
I	98.77											
II	96.92	99.49										
IIIb	96.25	96.97	99.68									
IV	95.96	96.59	96.12	99.62								
VI	96.54	96.97	96.27	96.02	99.67							
VII	94.6	95.3	95.07	96.96	94.83	99.57						
VIII	96.84	96.4	95.91	97.47	95.96	96.5	99.96					
IX	95.58	96.24	95.86	95.34	95.68	94.32	95.2	99.86				
X	96.42	96.96	96.69	95.96	96.4	94.97	95.95	96.05	99.35			
XI	96.64	98.11	96.92	96.51	96.79	95.1	96.2	96.12	96.87	99.17		
S. arizonae	94.56	94.89	94.8	94.54	94.49	94.13	94.71	94.02	94.66	95	99.61	
S. bongori	90.8	91.1	91.14	90.73	90.74	90.36	90.69	90.77	90.85	90.93	90.42	99.54

C) rMLST

	I	II	IIIb	IV	VI	VII	VIII	IX	X	XI	S. arizonae	S. bongori
I	99.8											
II	99.37	99.91										
IIIb	99.21	99.53	99.91									
IV	98.88	99.13	99.13	99.84								
VI	99.16	99.41	99.35	98.98	99.93							
VII	98.58	98.73	98.75	99.19	98.61	99.81						
VIII	99.08	98.98	98.97	99.42	98.85	99.02	99.8					
IX	98.76	98.94	98.89	98.8	98.78	98.47	98.78	99.94				
X	99.28	99.48	99.42	99.02	99.27	98.6	98.92	98.85	99.86			
XI	99.11	99.36	99.24	98.91	99.19	98.55	98.83	98.77	99.23	99.79		
S. arizonae	98.58	98.65	98.64	98.58	98.48	98.42	98.45	98.33	98.59	98.44	99.96	
S. bongori	98.16	98.36	98.42	98.11	98.38	98.03	98.05	98	98.33	98.23	98.02	99.94
