# Supplementary material for: An evaluation of the species and subspecies of the genus Salmonella with whole genome sequence data: Proposal of type strains and epithets for novel S. enterica subspecies VII, VIII, IX, X and XI
Source: Genomics. 2021 Sep;113(5):3152–62. doi: 10.1016/j.ygeno.2021.07.003 (PMC8426187; doi:10.1016/j.ygeno.2021.07.003)
Supplement: Supplementary Fig. 4 — a and b: ANI analysis using both randomly selected representatives of subspecies and type strain of (A) isolate SAL_BA7507AA and (B) isolate SAL_MA5841AA. ANI analyses of the isolates within this study which did not cluster consistently. These isolates were compared to representatives of the other subspecies. The results are presented in a heatmap from red – yellow, representing most to least related. A: ANI analysis comparing the WGS of SAL_BA7507AA with the WGS of representative isolates for each other species and subspecies. The values for SAL_BA7507AA when compared to subspecies II and XI were coloured red, indicating close relatedness. B: ANI analysis comparing the WGS of SAL_MA5841AA with the WGS of representative isolates for each other species and subspecies. Values for SAL_MA5841AA when compared to subspecies I scored in red. [file mmc4.pdf]

A

WGS_type

	I	II	IIIb	IV	VI	VII	VIII	IX	X	XI	S. arizonae	S. bongori
SAL_BA7507AA	96.07	97.65	96.08	95.44	96.01	94	95.46	95.07	95.94	97.69	93.69	89.78

WGS_random

	I	II	IIIb	IV	VI	VII	VIII	IX	X	XI	S. arizonae	S. bongori
SAL_BA7507AA	96	97.99	95.98	95.52	95.88	94.02	95.36	95.17	95.87	97.87	93.72	89.88

B

WGS_type

	I	II	IIIb	IV	VI	VII	VIII	IX	X	XI	S. arizonae	S. bongori
SAL_MA5841AA	99.89	96.08	95.14	94.86	95.59	93.67	95.8	94.37	95.19	95.7	93.42	89.84

WGS_random

	I	II	IIIb	IV	VI	VII	VIII	IX	X	XI	S. arizonae	S. bongori
SAL_MA5841AA	99.98	96.1	94.96	94.98	95.67	93.61	95.98	94.55	95.37	95.74	93.32	89.95
